# Plasma lipids and growth faltering: A longitudinal cohort study in rural Gambian children

**DOI:** 10.1126/sciadv.abj1132

**Published:** 2021-09-17

**Authors:** Gerard Bryan Gonzales, Daniella Brals, Bakary Sonko, Fatou Sosseh, Andrew M. Prentice, Sophie E. Moore, Albert Koulman

**Affiliations:** 1 Nutrition, Metabolism, and Genomics Group, Division of Human Nutrition and Health, Wageningen University, Stippeneng 4, Wageningen, 6708 WE, Netherlands.; 2 Laboratory of Gastroenterology, Department of Internal Medicine and Paediatrics, Faculty of Medicine and Health Sciences, Ghent University, 9000 Ghent, Belgium.; 3 Core Metabolomics and Lipidomics Laboratory, Wellcome Trust-MRL Institute of Metabolic Science, University of Cambridge, Cambridge, UK.; 4 Metabolic Disease Unit, Wellcome Trust-MRL Institute of Metabolic Science, University of Cambridge, Cambridge, UK.; 5 Department of Global Health, Amsterdam Institute for Global Health and Development, Amsterdam University Medical Centres, Amsterdam, Netherlands.; 6 MRC Unit The Gambia at London School of Hygiene and Tropical Medicine, Banjul, Gambia.; 7 Women & Children’s Health, King’s College London, London, UK.

## Abstract

Growth faltering in children arises from metabolic and endocrine dysfunction driven by complex interactions between poor diet, persistent infections, and immunopathology. Here, we determined the progression of the plasma lipidome among Gambian children (*n* = 409) and assessed its association with growth faltering during the first 2 years of life using the panel vector autoregression method. We further investigated temporal associations among lipid clusters. We observed that measures of stunting, wasting, and underweight are dynamically associated with each other and that lipid groups containing polyunsaturated fatty acids (PUFAs) and phosphatidylcholines consistently predict future growth outcomes. Linear growth was dynamically associated with the majority of lipids, indicating a higher nutritional demand to improve height compared to weight among growth-restricted children. Our results indicate a critical role for PUFAs and choline in early life dietary interventions to combat the child growth faltering still so prevalent in low-income settings.

## Teaser

Plasma lipids are associated with growth in the first 2 years of life among children at high risk of growth faltering.

## INTRODUCTION

The first 1000 days (from conception to 2 years of age) are critically important in determining individual health trajectories to adulthood, and exposures during this period—especially nutritional exposures—can have lasting negative impact (*1*). The causes of malnutrition are complex and multifaceted, involving the interplay between nutrition, hygiene, infections, maternal health, economic status, and other sociodemographic factors (*2*). Malnutrition, which here refers to undernutrition, is characterized by stunting or having a length-for-age *z* score (LAZ) below −2 SD, wasting or having below −2 SD weight-for-length *z* score (WLZ), and underweight or being below −2 SD weight-for-age *z* score (WAZ) (*3*). Stunting is believed to be a result of chronic nutrient deprivation (chronic malnutrition), whereas wasting results from short-term malnutrition (hence, often referred to as acute malnutrition). Underweight is a reflection of both wasting and stunting (*4*). Global estimates suggest that in 2019, 144 million children under 5 years of age were stunted, while 47 million were wasted (*5*), and this number is expected to rise due to the effect of the severe acute respiratory syndrome coronavirus 2 (SARS-CoV-2) pandemic (*6*).

Omics-based approaches have been used to gain a deeper understanding into the biochemical and metabolic perturbations that occur among children with malnutrition. However, the majority of these reports have focused on analyzing samples and data from cross-sectional studies (*7*–*10*); data from longitudinal studies are needed to help understand the timing and direction of associations. By following the metabolome and lipidome progression over time in a single individual, resolution is enhanced, because interindividual sources of variability [i.e., differences in (epi)genetic and lifestyle characteristics] are controlled. However, longitudinal analysis of high-dimensional data in field-based settings and among populations most at risk from undernutrition, especially metabolomics and lipidomics, remains challenging due to logistical and practical issues in field-based studies in low-resource settings. Furthermore, where longitudinal analyses exist, data analysis methods used have been limited to assessing the progression of metabolic features over time, ranking the most dynamic features (*11*–*15*), and not exploring potential causality or associations among the different metabolic features over time.

While the systems biology field has been exploring novel approaches to investigate longitudinal data and its association with specific clinical outcomes, other disciplines, such as econometrics and social sciences, have been analyzing the same types of problems using robust data analysis approaches backed by strong mathematical foundations (*16*–*20*). In our current study, we investigated the association between progression of the lipidome and growth outcomes in the first 2 years of life among children in The Gambia using an econometric-based causal inference approach applied to systems biology. Here, we adopt the panel vector autoregressive (PVAR) method in a generalized method of moments (GMM) framework to infer the directions of associations among serum lipids and growth outcomes in these children.

## RESULTS

### Population characteristics

A total of 1631 serum samples were analyzed from 409 individual children from 3 months of age up to 2 years (five time points). A total of 205 children had samples from all five time points, 77 from four time points, 63 from three time points, and the remainder (65) had samples from two time points (*21*). Table 1 highlights child characteristics by time point. In general, a decline over time in WAZ, LAZ, and WLZ was observed, indicating growth faltering in this population. Males had significantly lower WAZ, LAZ, and WLZ than females across the first 2 years, but their growth patterns were not different from each other (i.e., no interaction between sex and age was found, *P* = 0.70).

**Table 1.** Growth characteristics of 410 Gambian children in the first 2 years of life.

	**Age in weeks**					Trend*
	**12**	**24**	**52**	**78**	**104**	
*N*	298	327	323	345	338	
*N* girls (%)	138 (46.3)	155 (47.4)	157 (48.6)	165 (47.8)	158 (46.7)	
WAZ, mean ± SD	−0.70 (1.04)	−0.81 (1.17)	−1.26 (1.06)	−1.28 (1.06)	−1.38 (0.93)	β = −0.22
						*P* < 0.001
LAZ, mean ± SD	−0.37 (1.04)	−0.46 (1.03)	−1.03 (1.03)	−1.13 (1.05)	−1.33 (0.94)	β = −0.28
						*P* < 0.001
WLZ, mean ± SD	−0.52 (1.12)	−0.63 (1.22)	−1.03 (1.14)	−1.01 (1.07)	−0.97 (0.93)	β = −0.16
						*P* < 0.001

*Partial correlation (β) and *P* value obtained using fixed-effects panel model analysis, i.e., by estimating the following equation: *Y_it_* = α*_i_* + β*T*_t_ + ε*_it_*, where *Y_it_* is the respective growth parameter (WAZ, LAZ, or WLZ), α*_i_* is the individual fixed effect representing unobserved time-constant characteristics of the child, and *T*_t_ is the time-trend variable, which takes values between 1 (12 weeks) and 5 (104 weeks).

**Fig. 1.**
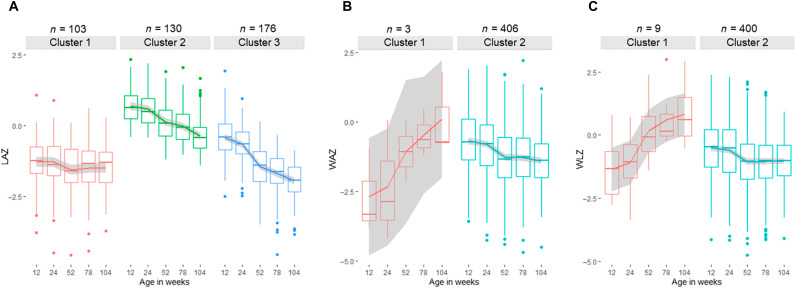
Growth patterns of children from 12 to 104 weeks of life. Clusters of similar growth curves were generated using latent class mixed modeling. (**A**) Three latent groups representing different LAZ progression in the population—25% belonged to cluster 1, 32% to cluster 2, and 43% to cluster 3. For WAZ (**B**) and WLZ (**C**), two latent groups were obtained, but 98 to 99% of the population belonged to cluster 2. A few children showed an increasing trend in their WAZ (three) and WLZ (nine) in the first 2 years of life.

Using latent class linear mixed models, we identified subclusters within the population characterized by different growth patterns in the first 2 years of life. For LAZ, we identified three patterns of growth (Fig. 1A). Cluster 1 (32%) included children who started with low LAZ at week 12 and remained at their low LAZ over time. Cluster 2 (43%) included children with the highest LAZ at week 12, which gradually decreased over time but did not drop below −2 SD, indicating that children in this cluster were not considered stunted as classified by the World Health Organization (WHO) definition. Almost half (49.7%) of the children belonged to cluster 3, which was characterized by midlevel LAZ at week 12 and having a steep decline in LAZ toward stunting over time. By week 104, 26% (25 of 95) of those in cluster 1 were stunted, whereas this was 47% (65 of 138) in cluster 3.

For WAZ (Fig. 1B) and WLZ (Fig. 1C), two clusters were identified, but 98 and 99% of the children belonged to the second cluster for WAZ and WLZ, respectively. Three children had WAZ of −2.68 ± 1.52 at week 12 but caught up in weight by week 104 (WAZ = 0.12 ± 1.47). In addition, the WLZ of nine children at week 12 (WLZ = −1.36 ± 1.20) had significantly increased by week 104 (WLZ = 0.82 ± 1.13) (*P* < 0.05). Individualized growth patterns are shown in fig. S1.

### Lipidome progression in the first 2 years of life

**Fig. 2.**
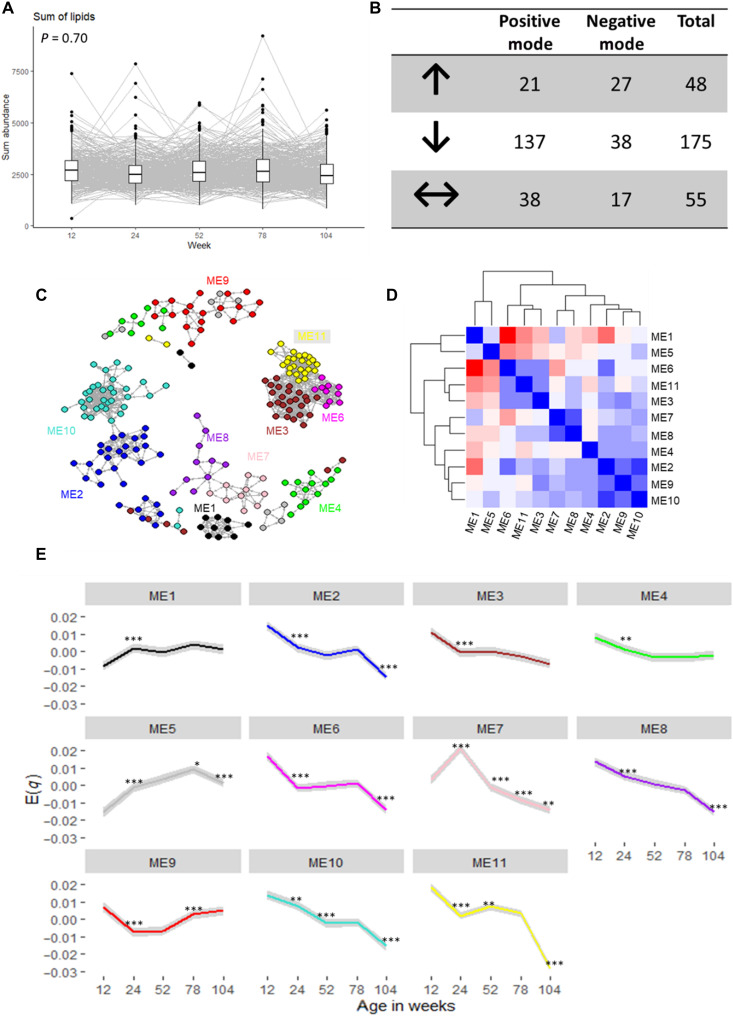
Lipid progression in the first 2 years of life among children in The Gambia. (**A**) Sum of total lipids over time. Fixed-effects panel analysis revealed no significant change in total lipids over time (*P* = 0.70). (**B**) Number of lipids significantly altering through time [*P* = 0.05 adjusted for false discovery rate (FDR)]; ↑ indicates significant increase, ↓ indicates significant decrease, and ↔ indicates no significant change after Bonferroni correction. (**C**) Weighted correlation network showing 11 lipid clusters obtained using the WGCNA package in R. (**D**) Intermodular relationship showing closely related lipid clusters (modules). (**E**) Progression of eigenlipid (ME*q*, where *q* is the module number), which represents the collective behavior of the lipids in the module, over time. Significance levels: ***P < 0.0001; **P < 0.001; *P < 0.01. Comparisons were made using paired *t* test comparing the time point with the preceding time point. Analysis was only made among those with values in both time points. Gray shadow around the line indicates the 95% confidence interval.

The total serum lipids (sum of all individual lipids) did not significantly change in the first 2 years of life, indicating that the lipid pool is conserved during infant growth (Fig. 2A). However, serum lipid composition appeared to change over time. The serum concentration of most lipids identified (175 of 278, 63%) significantly decreased over time, whereas 17% (48 of 278) had a significant upward trend. Several lipids (55 of 278, 30%), on the other hand, were conserved during the first 2 years of life (Fig. 2B). The progression of all identified lipids with age is shown in table S1.

To reduce the number of independent variables for succeeding analyses, we identified clusters of highly correlated lipids (“modules”) using weighted correlation network analysis (*22*). Lipids assigned to their respective modules potentially share similar physiological and molecular characteristics, as modules reflect functional relationships (physical and nonphysical interactions) among its members (*22*). Each module is characterized by an eigenlipid (ME*q*, where *q* denotes the module), which is a unique representation that most closely reflects the collective behavior of the module (*23*). This indicates that the progression of lipids in each module over time is reflected by the dynamics of the ME*q*. About 87% (241 of 287) of the lipids were clustered into 10 modules, whereas the remaining 37 (13%) lipid species were unassigned (gray module, ME5). Module assignment of all lipids is detailed in table S2. To obtain an overview of the interlipid correlations, we plotted the module correlation network (Fig. 2C), which shows that several modules are more closely correlated, creating bigger clusters of lipids as depicted on a heatmap showing hierarchical clustering (Fig. 2D).

The weighted correlation network analysis clustered lipids with very similar chemical or biological characteristics into different modules (Table 2). Most notably, triglycerides (TGs) with polyunsaturated (*n* > 5) fatty acid (PUFA) side chains (ME6) were clustered differently from shorter-chain TGs (ME11) and TG with PUFA containing fewer double bonds (*n* < 4). The most abundant phosphatidylcholines (PCs) found in serum were clustered in ME9, whereas cholesterol esters and sphingomyelins were clustered in ME10. Cholesterol esters and PCs with PUFA side chains, however, were clustered in a different module (ME2). LysoPCs with saturated FA (ME7) were also clustered differently from lysoPCs with PUFA (ME8). Oxidized PCs and ether-linked PCs were clustered in ME1. Last, any lipid that did not belong to any other module was clustered in ME5. However, these lipids also shared common characteristics such that this module is composed of free FAs and FA oxidation products and their esters. Therefore, this module cannot be discounted. Each module had a characteristic progression from 12 to 104 weeks of infant age, where the biggest changes occurred between week 12 and week 24 (Fig. 2E).

**Table 2.** Association between module eigenlipid (ME*q*) and growth outcomes over time in the first 2 years of life. Upper numbers are partial coefficients estimated by using a fixed-effects panel model; lower numbers in parenthesis are FDR-adjusted *P* values. Boxes are colored blue when a significantly positive association was found and red when negative. Fixed-effects panel models were estimated by the following equation: *Y_it_* = α*_i_* + β*T*_t_ + ME*_it_* + ε*_it_*, where *Y*_it_ is the respective growth parameter (WAZ, LAZ, or WLZ), α*_i_* is the individual fixed effect representing unobserved time-constant characteristics of the child, *T*_t_ is a time-trend variable taking values between 1 (12 weeks) and 5 (104 weeks), and ME*_it_* is the respective module eigenlipid. PC, phosphatidylcholine; PS, phosphatidylserine; PE, phosphatidylethanolamine; TG, triglycerides; DG, diglycerides; FA, fatty acid; SFA, saturated FA; PUFA, polyunsaturated FA; MUFA, monounsaturated FA; PA, phosphatidic acid.

**Module**	Size*	**Outcomes**			**Main composition**
		WAZ	LAZ	WLZ	
ME1	16	−2.65	−0.06	−3.67	Ether-linked PCs and PSs, oxidized PCs
		(<0.001)	(0.92)	(<0.0001)	
ME2	39	2.55	0.58	2.66	All PUFA-containing lipids [both *n*-3 (22:6, 20:5) and *n*-6 (20:4)], the cholesterol esters, PCs, PC-O/PC-PE-O/ PE-P
		(<0.001)	(0.54)	(0.011)	
ME3	31	−1.36	−1.47	−1.49	Most common TGs and DGs
		(0.14)	(0.19)	(0.22)	
ME4	26	0.13	−0.08	0.47	PA, PEs
		(0.84)	(0.92)	(0.70)	
ME5	37	−2.43	−0.34	−3.67	Unassigned lipids; free FAs and FA oxidation products and their esters
		(<0.001)	(0.71)	(<0.0001)	
ME6	11	1.26	−0.59	1.41	TG containing PUFAs
		(0.15)	(0.54)	(0.22)	
ME7	14	0.26	0.76	0.09	LysoPC mainly SFA and MUFA (sn1)
		(0.74)	(0.50)	(0.91)	
ME8	11	−0.89	−1.38	−0.82	LysoPC mainly MUFA and PUFA (sn2)
		(0.27)	(0.20)	(0.51)	
ME9	25	0.83	−0.64	1.45	Most abundant PCs
		(0.27)	(0.54)	(0.22)	
ME10	41	1.13	1.25	0.38	Most common cholesterol esters and sphingomyelins
		(0.18)	(0.19)	(0.74)	
ME11	27	−0.47	−1.29	−1.18	All small TGs and in-source fragments and isotopes
		(0.63)	(0.20)	(0.33)	

*Number of lipids belonging to the module.

#### Lipids associated with LAZ

Adjusting for age, we did not find any ME*q* that significantly associated with LAZ over time. However, associations did appear when age in weeks (included in the model as a time trend) was removed. This indicates that the eigenlipids were significantly associated with other factors changing through time but not with LAZ itself.

**Fig. 3.**
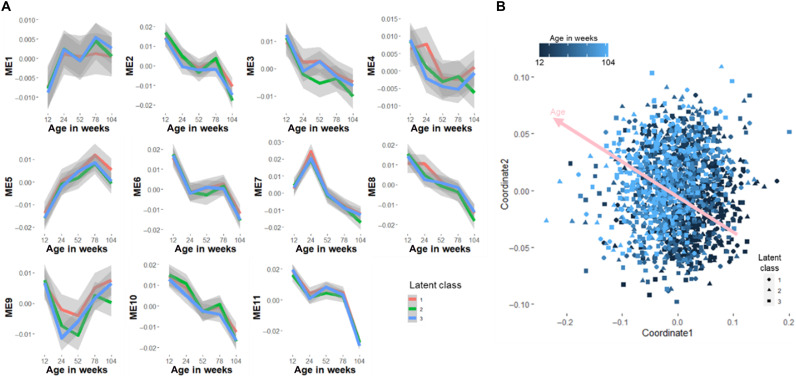
Eigenlipid progression of the children grouped based on latent class linear mixed modeling. (**A**) Each facet represents a module obtained from weighted correlation network analysis. No significant differences in the time course progression of ME*q* were observed among the three clusters in all modules. (**B**) Multidimensional scaling analysis showing time-dependent clustering of observations but no distinction between latent classes

We also did not find any significant differences in the ME*q* progression over time among the three LAZ clusters, indicating that general lipid progression is similar in all the children in this population through the first 2 years of life (Fig. 3A). Multidimensional scaling analysis shows that ME*q* induced a time-dependent clustering of the observations, but no latent class-specific clustering is evident (Fig. 3B). Analysis of individual lipid species instead of ME*q* also showed that lipid progression was not dependent on the LAZ growth trajectory (table S3).

#### Lipids associated with WAZ and WLZ

WAZ and WLZ were highly correlated at all time points (*r* = 0.83, *P* < 0.001). Consequently, similar modules are associated with these anthropometric measures. Adjusting for age, serum levels of oxidized and ether-linked PCs (ME1) and free FAs (ME5) tend to have an opposite trend with WAZ and WLZ progression (*P* < 0.001). Conversely, the progression of PUFA-containing lipids (ME2) tended to have the same trends as WAZ and WLZ (*P* < 0.001) over time. As highlighted, almost all children followed the same WAZ and WLZ growth trajectory, except for a very small number of children who have improved growth parameters over time. Hence, we did not compare differences in lipid progression between these WAZ and WLZ latent classes, as there would not have been enough observations in the first cluster to make a reliable comparison.

### PVAR model using system GMM approach

**Fig. 4.**
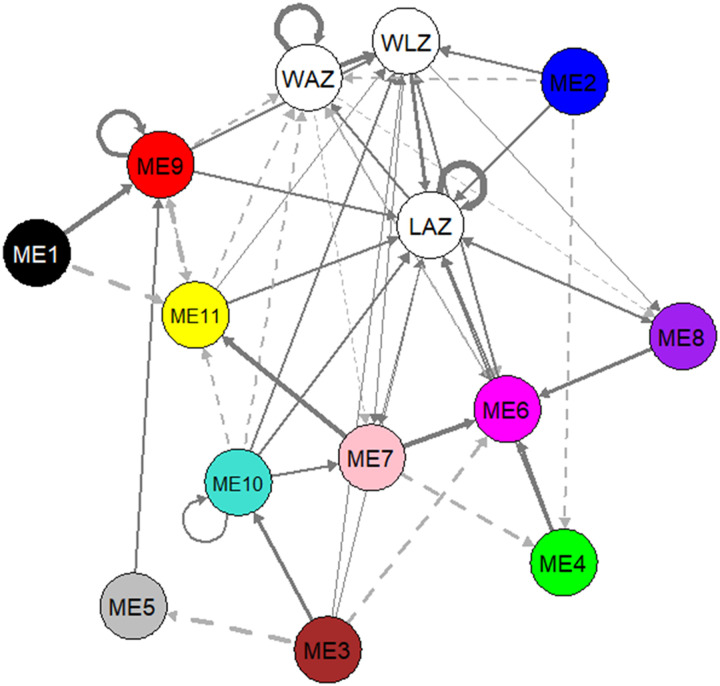
Results of system GMM-PVAR analysis. Temporal network visualization of the system GMM-PVAR model. Arrows indicate that a node predicts another node (or itself) in the next time point. Full arrows indicate positive association, while dashed arrows indicate negative association. Loops indicate that the current value of a node predicts the future value of itself. Arrow thickness depicts the strength of the association. Node annotation for ME1 to ME11 is shown in Table 2. All roots are inside the unit circle indicating stability of the model and stationarity of the variables (fig. S2). WAZ, LAZ, WLZ, and all ME eigenlipids were included in the model as endogenous variables in the first order (lag *t*−1). Sex variation was taken into account by adjusting for sex as an exogenous variable.

We assessed the associations among plasma lipids and growth outcomes using dynamic panel data analysis, specifically PVAR model, which is an econometrics-based causal inference method. A first-order PVAR model (lag *t*−1) was selected as optimal lag length based on the model selection procedure of Andrews and Lu (*17*). Table S4 shows the estimates of the system GMM-PVAR model for the three growth outcomes and 11 eigenlipid modules. We visually represented the GMM-PVAR model results as a temporal network as shown in Fig. 4. In this temporal network, current (*Y_t_*) and lagged values (*Y*_*t*−1_) of growth outcomes and each lipid module are combined into individual nodes, which are connected with directed edges according to the regression estimates of the model (table S4, see “Coef”). The direction of the arrows indicates that current values of a node are consistently associated to the next (*t* + 1) value of the other node, or with itself in case of a loop. Full arrows indicate a positive association, while dashed arrows indicate a negative association, and arrow thickness gives the strength of the association. Only significant (*P* < 0.05) associations are shown, which mean that the observed association is consistent for every succeeding time point. Such association shall henceforth be referred to as *G* associations here. Hansen test for overidentifying restrictions did not reject the null hypothesis, implying that all instruments used are valid. The stability of the PVAR was confirmed as the eigenvalues are strictly less than 1, and none of the roots are outside the unit circle (fig. S2), indicating that the model is stable and also that our variables are stationary (*24*, *25*).

The results indicate that the growth parameters are *G* associated with each other to varying degrees; gains in WAZ are associated with increased future WLZ and WAZ itself, whereas gains in WLZ are associated with an increase in LAZ. Length growth is also positively *G* associated with weight. Modules consisting of PUFA-rich lipids (ME2 and ME6), PCs (ME2 and ME9), TGs (ME2 and ME6), and cholesterol esters (ME2 and ME10) are positively *G* associated with WLZ. However, these same lipids have negative *G* associations with WAZ in a population with high burden of growth faltering.

Most lipid modules had positive *G* associations with LAZ, indicating that more biological processes and building blocks are potentially demanded to increase height rather than weight. In addition to the lipids dynamically associated with WLZ, LAZ is also *G* associated with serum levels of phosphatidic acid and phosphatidylethanolamine (ME4) and lysoPC containing monounsaturated FA (MUFA) and PUFA lipids (ME8). ME9 had the biggest positive *G* association with LAZ and WLZ among all the lipids. These positive *G* associations indicate that overall changes in the serum levels of these lipids will likely induce a change in LAZ and WLZ in the same direction; increasing serum levels of these lipid groups may lead to an increase in LAZ and WLZ.

Ether-linked PCs (ME1), free FAs (ME5), saturated FA (SFA)/MUFA-lysoPCs (ME7), and small TGs (ME11) did not show significant *G* association on any of the growth parameters. However, these lipids were shown to be *G* associated with the levels of the other lipids. For instance, levels of ME1 and ME5 predict increased ME9 levels, whereas ME11 predicts decreased ME9. Increasing ME7 is associated with a decrease in ME5 but an increase in ME11. The interplay of the lipids indicates the dynamic interactions, including synthesis and oxidation cycles, that occur between the lipid groups.

## DISCUSSION

In this study, we introduced a statistical technique typically used in econometrics and social sciences to infer *G* associations among growth outcome parameters and plasma lipids in the first 2 years of life of African children living in an area with a high burden of growth faltering. Using the PVAR method, we showed which lipids are *G* associated with growth and also how different lipids may influence each other over time.

We first characterized the children’s growth patterns in clusters using latent class mixed modeling. Previous studies that have compared growth parameters in children with metabolites have typically characterized children as either growth impaired (e.g., stunted or underweight) or healthy, and determined which metabolites or lipids are able to classify them based on this binary classification (*7*, *26*) even for studies that observed children over a period of time (*14*). Using latent class modeling, we showed that the children from rural Gambia experienced a general decline in growth outcome over time, albeit at different trajectories. This observed growth faltering has been reported previously for this cohort of children (*27*, *28*) and is also observed to be common among children in low- and middle-income countries (LMICs) (*29*). However, while some children remained stunted over the first 2 years of life, some children remained above the stunting cutoff (despite reduced growth), while others started as normal and slowly faltered ending up as stunted in the longer term. This indicates that a binary classification (impaired versus healthy) for growth faltering does not adequately capture the growth trajectories in these children.

For weight measures (WLZ and WAZ), we observed a general decline, except in a very small number of children who increased in WAZ/WLZ over time, which makes statistical comparison difficult. Future studies to investigate the progression of serum lipids among those with different WAZ/WLZ trajectories therefore require a much bigger sample size to capture enough number of children in both groups.

A number of studies have followed the metabolic status of children through the early years of life, especially in the first 2 years (table S5) (*11*–*15*). However, most of these studies focused on well-nourished populations. A notable exception to this was the study by Giallourou *et al*. (*14*) that followed the changes that occur in the metabolome of children by analyzing urine and plasma samples at 3, 6, 9, 15, and 24 months of age among children in three resource-constrained countries (Peru, Bangladesh, and Tanzania) (*14*). The authors used a phenome-for-age *z* score (PAZ) and found that PAZ of stunted children lagged compared to healthy children, indicating poor metabolic maturity. These studies mainly used linear mixed/multilevel models, analysis of variance (ANOVA), and other multidimensional data analysis techniques [principal components analysis, partial least squares regression, and ANOVA–simultaneous component analysis (ASCA)]. Although associations with growth outcomes can be deduced using these methods, they typically do not show variable interrelatedness and do not assess potential causal links between the metabolome/lipidome and growth outcomes.

Similar to Nikkilä *et al*. (*15*), who studied serum lipidome progression among Finnish children from birth to 2 years of age, we began by clustering tightly correlated lipids into modules to reduce dimensionality. To do this, we used weighted gene correlation network analysis (*22*). We observed that the algorithm clustered lipid species based on their chemical features, specifically type of lipid species, length, and (un)saturation (SFA, MUFA, and PUFA). These clusters indicate that serum levels of these lipids behave very similarly across time in the population, enabling us to generalize their association with growth outcomes. We observed that major metabolic changes occurred around the first 6 months of life, which corresponded to the start of the transitional feeding in our study population—when children started taking other foods apart from breast milk. In this population of infants, rates of exclusive breast feeding (EBF) are high, with a mean duration of EBF across the whole of the study cohort of 5.2 months (*27*).

Here, we analyzed longitudinal biochemical data using panel data analysis methods from the field of econometrics. Panel data are a hybrid of cross-sectional and time series data, where data are collected for *N* individuals over *T* occasions, which is typically the most common design used in longitudinal systems biology studies. Systems biology can benefit from panel data analysis, as it is best suited for studies with large *N* but small *T*, which is most common especially in clinical studies where participants are not able to provide numerous biological samples over long periods of time. To the best of our knowledge, this paper is the first to demonstrate the use of panel data analysis methods in systems biology.

Despite observing three categories of LAZ trajectories, we found no significant differences in the serum lipid profiles of these children over time, which concurs with our basic panel analysis (Table 2). Contrary to LAZ, we observed that lipolysis products (free FAs and oxidized PCs) progress reciprocally to the progression of WAZ and WLZ. However, this association does not imply that free FAs and oxidized PCs cause the decline in WAZ and WLZ in the first 2 years of life. One requirement for establishing a causal relationship is to demonstrate consistent significant association between current levels of the exposure and future levels of the outcome. One main advantage of using dynamic panel analysis strategies is its ability to infer potential causal links between outcomes and variables in a longitudinal study (*30*). In econometric terms, causality is assessed in terms of Granger causality, which is a statistical concept based on prediction. Under this framework, a time series (ME*q*) is Granger causal of another time series (LAZ) if inclusion of the history of (ME*q*) improves prediction of LAZ over knowledge of the history of LAZ alone (*31*). This is achieved by incorporating lagged (*t*−1…*n*) values as independent variables in the model, which will lead to biased estimates when performed using ordinary linear regression models due to the Nickell bias (*20*). This bias is eliminated by using GMM in dynamic panel analysis (*16*, *18*, *19*, *32*). Granger causality is not only the most adopted criterion for causal inference in economics but also used in other fields such as neuroimaging (*33*). However, as our study is an observational clinical study, causality cannot be fully concluded in epidemiological terms using econometric tools alone. For this reason, we referred to these associations as *G* associations instead of causal pathways. The main advantage of our analysis is therefore that it provides an insight into which specific lipid targets could be tested further in randomized control trials (RCTs). This strategy increases the likelihood of success of the RCT, where the actual causal link will be tested.

In this study, we used a system GMM-PVAR model, which allowed us to simultaneously assess the *G* associations between serum lipid profile and growth outcomes, and also how different lipid species are consistently associated with each other over time. The PVAR model is a modification of the conventional VAR model, which deals with panel data that typically comprise designs with *N* > *T* (*34*). PVAR also addresses individual heterogeneity from each individual cross-sectional unit (in this case, each child) (*34*). Hence, using this method, we are able to establish *G* associations and also assess variable interrelatedness, which previous longitudinal studies in children fail to report.

Our results suggest a *G* association between being underweight to being wasted and subsequently stunted. In a compilation of datasets from 1.8 million children in 51 countries, it was previously reported that all children that were both stunted and wasted were also underweight (*35*), indicating a cross-sectional association among the three growth parameters. However, it has also been previously demonstrated that wasting precedes stunting and children with low WLZ were at a higher risk of linear growth retardation (stunting), especially for those below 3 years old (*36*–*38*). Wasting at younger age (from 6 to 17 months) was associated with stunting from 18 months of age. This association was, however, not observed when wasting occurred below 6 months of age (*36*, *37*). These earlier reports indicate that the association between the three growth outcomes was accurately captured by the PVAR model, indicating the validity of our approach.

It may, however, seem counterintuitive that although WAZ and WLZ are highly correlated (Table 2), directions of *G* associations with MEs are opposite from one another (Fig. 4). However, we need to consider that the results should be viewed as a system and not as individual independent nodes. Note that, despite being statistically correlated, WAZ and WLZ are not the same. Our analysis highlights that the physiology that results in the growth parameters is different.

WAZ is weight. Future weight of the child is positively influenced when current weight of the child, TGs (ME6), and height (LAZ) are high, whereas levels of other lipids, especially PUFA-rich ones (ME2; 8 and 11), PCs (ME9), and cholesterol esters and sphingomyelins (ME10), are low. The effect of TGs on weight is well known, whereas an increase in height would naturally increase weight. Furthermore, in a European study, full-term infants fed a higher level (3.2%) of α-linolenic acid (ALA) during the first 4 months of life had higher plasma levels of docosahexaenoic acid (DHA) and lower mean group weight than infants on a 0.4% ALA formula (*39*). These results concur with our PVAR model, indicating a negative causal link between PUFA-rich lipids (ME2, ME8, and ME11) and WAZ.

WLZ is a measure for wasting (in these children), and hence a proxy indicator of lean mass. Our results indicate that current weight is a large driver of future wasting status, but increasing weight alone by providing a child with TGs will not be enough to increase lean mass. To increase future WLZ, these PUFA-rich lipids are needed on top of increased weight. Our analysis therefore highlights the need for deeper statistical analysis such as the use of econometric tools (i.e., PVAR in Fig. 4 and table S4), as these important information would be completely missed when only relying on correlations (Table 2).

In the interpretation of the lipid data, it is important to understand that circulating lipids in the first years of life play a crucial role in the growth and development of many vital organs, most of all the brain, which requires lipid for growth and myelination. However, most information that we have in the literature is still mainly limited to European or other high-income settings. Our results demonstrated that in this population, the majority of lipids contributed to LAZ, indicating higher energy and biochemical requirements for increasing linear growth than increasing weight. WLZ alone is insufficient to influence future LAZ, and several lipid clusters are needed to improve LAZ. This shows that more factors are associated with stunting than is explained by prior wasting, as also previously hypothesized (*36*). The different classes of lipids involved indicate that it is not only the lipids that provide energy that are limiting growth. Most notably, lysoPCs composed of MUFA and PUFA (ME8) were exclusively positively causal to LAZ compared to WLZ and WAZ. Evidence on the effect of PUFA, especially DHA, prenatal supplementation on infant height has been inconsistent (*40*). In one study, prenatal DHA supplementation resulted to a significant increase in infant height at age 18 months compared to placebo (*41*), but this effect was no longer observed when the children were followed to 60 months of age (*42*). Moreover, cord blood PUFA levels were found to have a sex-specific association with infant height at 6 months of age, where *n*-3 PUFA levels were associated with higher infant length in males, while higher *n*-6 PUFA concentrations were associated with lower length in infancy. However, higher cord blood *n*-3:*n*-6 ratio was associated with higher infant length at 6 months of age. These associations were, however, no longer observed at later time points (from 2 years of age) (*43*). It is important to interpret these results in relation to nutrient availability. Brain development and growth requires large amounts of PUFAs, as the brain’s lipid composition comprises 35% PUFAs, which cannot be synthesized de novo (*44*). Hence, insufficient PUFA intake may require the body to use energy for FA desaturation to enable brain development and growth, limiting the energy available for lateral growth. Supplementation with PUFAs can therefore have very different effects on growth, depending on the availability of other nutrients. Hence, the potential effect of PUFA on LAZ may not be consistent. For instance, we have previously shown that PUFA supplementation did not improve growth and cognitive function of breast-fed infants in The Gambia, despite increasing plasma PUFA levels (*45*). PUFA intake was therefore not the limiting factor.

Of all lipids contributing to LAZ, PCs (ME9) had the highest *G* association on LAZ and WLZ. A metabolomics study reported reduced urinary levels of betaine and dimethylglycine, which are endogenous choline metabolites, in stunted Brazilian children, indicating possible reduction of choline bioavailability from the diet (*26*). Choline is an essential nutrient and is a precursor for PCs. Low serum choline was also previously reported to be associated with linear growth failure among children in Malawi (*46*). Eggs, particularly the egg yolk, are one of the main sources of dietary choline (*47*). Clinical trials using egg supplementation reported improved LAZ and height gain among children in Ecuador (*48*) and Uganda (*49*), respectively. Although eggs contain many other important nutrients, our data suggest that this efficacy could be due, at least in part, to the increase in intake of PC precursors.

Our current results demonstrate that all lipid species containing PUFAs (ME2, ME6, and ME8) and PCs (ME2 and ME9) were positively *G* associated to infant LAZ in the first 2 years of life. This underlines the importance of availability of essential lipids in early life nutrition in these populations. This highlights the need to use evidence from studies in the target populations, rather than relying on evidence of just European studies. Growth faltering among children in LMICs occur at a population level (*29*), which indicates the need to study its determinants at a community level instead of looking at individuals. As the majority of these children in our study were exclusively breastfed until 5 months of age, poor maternal breast milk lipid composition could be an underlying factor associated with growth faltering. A survey of breast milk composition from mothers in area with high burden of infant growth faltering is therefore warranted and could be a target for intervention.

Furthermore, environmental factors potentially contribute to the malabsorption of PUFAs and choline in these children. Environmental enteric dysfunction is a subclinical state of intestinal inflammation commonly observed in children in LMICs (*50*), which may affect absorption of these lipids from breast milk. Hence, efforts to improve sanitation and reduce incidence of infections in children may improve bioavailability of essential lipids, which leads to improved growth outcomes.

Although this paper greatly contributes to the very limited data available on the interaction between lipids and growth outcomes in the first 2 years of life, we acknowledge that our study would be improved if children with more variable growth trajectories were included. In this study population, most children exhibited very similar growth trajectories and were growth impaired, especially stunted. Future studies involving children with different growth outcomes within the same population is therefore warranted.

In this study, we used a high-throughput lipidomic method, which does not provide a more thorough lipid identification compared to liquid chromatography–mass spectrometry (LC-MS)–based techniques. However, the weighted correlation network analysis allowed us to cluster lipids with similar structural and biochemical properties, which compensates for the lack of specificity in individual lipid identifications.

The link between underweight and wasting to stunting indicates that measures and interventions to address childhood stunting may require prevention of underweight and wasting earlier in life. Demonstrating the role of circulating lipids in growth regulation among infants in low-resource areas offers insights into potential intervention strategies based on nutritional formulation with specific lipid compositions or those that trigger increase in circulating levels of specific lipid species, especially PUFAs and PCs.

## MATERIALS AND METHODS

### Study population

The analyses presented included data and samples collected as part of the Early Nutrition and Immune Development (ENID) study, a randomized trial conducted in the rural West Kiang region of The Gambia between April 2010 and February 2015. The full ENID trial protocol is described by Moore *et al*. (*21*), and the trial was registered as ISRCTN49285450. Briefly, mother-infant pairs were recruited in pregnancy (<20 weeks of gestation) and followed until 2 years post-partum. During pregnancy, women were randomly assigned to four trial arms, comparing combinations of protein-energy and multiple micronutrients, and from 6 to 18 months of age, their infants received either a daily multiple micronutrient-enriched lipid-based nutritional supplement (LNS) or a placebo LNS. As part of the trial design, infant anthropometry and blood samples were collected at clinic visits at 12, 24, 52, 78, and 104 weeks of infant age. Full details of measurement and sample collection protocols can be found in the trial protocol (*27*). The analyses presented here were not planned in the original study design and used data and samples from the first 400 infants born into the ENID trial.

The ENID trial was approved by the joint Gambian Government/MRC Unit The Gambia Ethics Committee (projects SCC1126v2 and L2010.77). Written informed consent was obtained from all the participants before enrolment.

### Untargeted lipidomic analysis

Serum samples were stored at −80°C until assay. Lipids were extracted as described previously (*51*). Briefly, 100 μl of LC-MS–grade water and 150 μl of internal standard mix were added to 15 μl of serum in a 96-well glass-coated plate before mixing for 10 s. Subsequently, 750 μl of LC-MS–grade methyl-tertiary butyl ether and a further 200 μl of LC-MS–grade water were added to each well before shaking for 10 s. Once mixed, plates were spun at 845*g* for 2 min to achieve phase separation, with 25 μl of the upper organic phase transferred to a new glass-coated plate with 90 μl of MS mix (7.5 mM ammonium acetate in isopropanol:CH_3_OH 2:1), which was subsequently added to each well.

### Direct injection mass spectrometry (DIMS) lipidomic profiling

Samples were infused into an Exactive Orbitrap (Thermo Fisher Scientific, Hemel Hempstead, UK) using Triversa NanoMate (Advion, Ithaca, USA). Data collection began 20 s after the infusion began, initially analyzing samples in the positive ionization mode with an ionization voltage of 1.2 kV applied. Data were acquired between 150 and 2000 mass/charge ratio (*m*/*z*) with a scan rate of 1 Hz, giving a mass resolution of 65,000 at 400 *m*/*z*. A more detailed description of the instrument parameters can be found in the study of Harshfield *et al*. (*51*).

### Processing lipidomic data

Raw data files were converted to .mzXML files using msConvert (ProteoWizard) (*52*) and were subsequently processed in R (version 3.2.2) using an in-house script to compare spectra against a list of 1649 lipid species, with a relative intensity and mass deviation value recorded for each lipid in every sample. We applied four filtering steps for quality control of the data and focus subsequent analysis on analytically robust signals. The first step was to remove lipids with a mean mass deviation between expected and recorded mass of greater than 5 parts per million (ppm). The second step was to remove signals with an average intensity in the samples less than five times greater than in the blanks. The third step was to remove signals with 0 values in greater than 10% of samples. The final step was to remove lipids with *r* < 0.9 in our quality control (QC) dilution series.

### Data analysis

#### Analysis of growth outcomes

The changes in WAZ, LAZ, and WLZ over time were determined using a fixed-effects panel model specification

$$Y_{\mathrm{it}}=\alpha_{i}+\beta_{1}{\text{age}}_{\mathrm{it}}+u_{\mathrm{it}}$$ (1)

where α*_i_* (*i* = 1…*n*) is the individual fixed effect; *Y_it_* is either WAZ, LAZ, or WLZ; age*_it_* is the age of child *i* at age *t*; and *u_it_* is the error term. This was implemented using the plm package (*53*) in R (version 3.6).

We subsequently clustered the children based on their growth patterns using latent class mixed modeling implemented using the lcmm package (*54*). LAZ, WAZ, and WLZ values of the children at all time points were used as dependent variables, while sex and age were independent variables. Missing measurements were considered missing at random, and hence, children with incomplete measurements were included. Age and child ID were used as random effects to allow varying intercepts and slopes per individual time series. A five-quantile spline function was used for estimation. The number of latent classes was tested between 2 and 4, and model selection was based on the Akaike information criterion (AIC). For estimating LAZ, a three-latent class model yielded the least AIC value, whereas a two-latent class model yielded least AIC values for both WAZ and WLZ.

#### Correlation network analysis

To reduce data complexity, clusters of tightly correlated lipids were determined using weighted coexpression network analysis (WGCNA) (*22*). Scale-free topography typical of biological networks (*r*^2^ ≳ 0.8) (*23*) for our data was achieved using β = 18 for a signed network. A Pearson correlation (*s_ij_*) matrix was then generated between each lipid pairs (*i* and *j*), which was transformed into an adjacency matrix through the power transformation

$$\mathrm{aij}={\left(\frac{1+s_{\mathrm{ij}}}{2}\right)}^{\beta }$$ (2)

This power transformation punishes weak and negative correlations while amplifying strong positive correlations. As this study aimed to determine the dynamic changes in lipids over time, a signed network was used to determine lipids that move in the same direction over time. Using hierarchical clustering embedded with the WGCNA package, tightly correlated lipids are clustered into modules using the blockwiseModules function, setting the minimum number of lipids forming a module to 10. The network was visualized using igraph (*55*).

#### Association between modules and growth outcomes

Each member of the module is characterized by an eigenlipid (ME*q*, where *q* denotes the module number) through a singular value decomposition. ME*q* represents the collective behavior of the particular module (*23*). The progression of individual lipids or module ME*q* in the first 2 years of life was assessed using the fixed-effects panel model as in Eq. 1 but with ME*q* as additional independent variable

$$Y_{\mathrm{it}}=\alpha_{i}+\beta_{1}{\text{age}}_{\mathrm{it}}+\beta_{2}{\text{ME}q}_{\mathrm{it}}+u_{\mathrm{it}}$$ (3)

where ME*q_it_* indicates the ME*q* of child *i* at time *t*. Significant associations of growth outcomes and lipids through time were detected using *P* < 0.05 after adjusting for false discovery rate (FDR) (*56*).

#### Panel vector autoregression model

A PVAR model uses lags of the endogenous variables and analyzes interdependencies among variables of interest (LAZ, WAZ, WLZ, and 11 lipid modules obtained from the weighted correlation network analysis). We thus estimated a 14-variate PVAR model of order *p* with panel-specific fixed effects represented by the following equation

$$Y_{\mathrm{it}}={\sum_{l=1}}^{p}A_{l}y_{i,t-l}+v_{\mathrm{it}}+e_{\mathrm{it}}$$ (4)

where *Y_it_* is a (1 × 14) vector of endogenous variables for the *i*th cross-sectional unit (child) at time *t*, *y*_*i*,*t-l*_ is a 14 × 1 vector of lagged endogenous variables (*l* being the number of lags), and *v_it_* and *e_it_* are (1 × 14) vectors of dependent variable–specific fixed effects and idiosyncratic errors, respectively. *A_l_* represents the 14 × 14 matrix of endogenous parameters to be estimated. Sex was additionally included as an exogenous variable, but we did not adjust for randomization arm in the original clinical trial. This is because the trial included lipid-based nutrient supplements, which directly influenced the lipids in plasma. Hence, adjusting for randomization arm in the trial to test the association between lipids and growth is not appropriate because the trial is part of the pathway from exposure to outcome and controlling for it will block some of the effect (*57*).

Following the procedure of Sigmund and Ferstl (*25*), we used unbalanced panel data and estimated PVAR models by fitting a multivariate panel regression of each dependent variable on lags of itself using GMM. GMM specification requires stationarity, which means that all unit roots of the PVAR model should fall inside the unit circle.

The PVAR model was specified by first specifying the maximum lag order of the model using the method described by Andrews and Lu (*17*). Because of maximum *t* = 5, we only tested for either first-order (*t*−1) and second-order (*t*−2) panels. Lag selection was based on the AIC and Bayesian information criteria. Then, a first difference and system GMM approaches with either first difference or forward orthogonal deviation (fod) transformation were assessed. The stability of the model was then tested. The system GMM model with fod transformation yielded a stable model and was hence used in the final analysis. The PVAR model was generated using the package panelvar in R (*25*).

## Notes

View/request a protocol for this paper from *Bio-protocol*.
